# Supplementary material for: Content-rich biological network constructed by mining PubMed abstracts
Source: BMC Bioinformatics. 2004 Oct 8;5:147. doi: 10.1186/1471-2105-5-147 (PMC528731; doi:10.1186/1471-2105-5-147)
Supplement: Additional File 5 — The original Chilibot query results of the term "long-term potentiation (LTP)" and 22 other terms, limiting the latest references analyzed to the years 1990, 1995, 2000, and 2004. [file 1471-2105-5-147-S5.bz2 › chilibotAdditionalFile5/ltp2000/html/CREB_ACTIN.html]

 


 **CREB** and **ACTIN** 
  
Found 2 abstracts in PubMed,  **2 abstracts were retrieved and analyzed**.  


---

 Search Google  |
 PDF files only 
|  EDU domain only 

---

- Curr Biol, 1997   **A kinase anchor protein 75 increases the rate and magnitude of cAMP signaling to the nucleus.** .
  A kinase anchor protein 75 AKAP75 binds regulatory subunits RIIalpha and RIIbeta of type II protein kinase A PKAII isoforms and targets the resulting complexes to sites in the cytoskeleton that abut the plasma membrane 1 7 .
  Co localization of AKAP75 PKAII with adenylate cyclase and PKA substrate effector proteins in cytoskeleton and plasma membrane effects a physical and functional integration of up stream and downstream signaling proteins, thereby ensuring efficient propagation of signals carried by locally generated cyclic AMP cAMP 4 9 .
  An important, but previously untested, prediction of the AKAP model is that efficient, cyclic nucleotide dependent liberation of diffusible PKA catalytic subunits from cytoskeleton bound AKAP75 PKAII complexes will also enhance signaling to distal organelles, such as the nucleus.
  We tested this idea by suing HEK A75 cells, in which PKAII isoforms are immobilized in cortical cytoskeleton by AKAP75.
  Abilities of HEK A75 and control cells with cytoplasmically dispersed PKAII isoforms to respond to increases in cAMP content were compared.
  Cells with anchored PKAII exhibited a threefold higher level of nuclear catalytic subunit content and 4 10 fold greater increments in phosphorylation of a regulatory serine residue in cAMP response element binding protein **CREB** and in phosphoCREB stimulated transcription of the c fos gene.
  Each effect occurred more rapidly in cells containing targeted AKAP75 PKAII complexes.
  Thus, anchoring of PKAII in **actin** cortical cytoskeleton increases the rate, magnitude and sensitivity of cAMP signaling to the nucleus.

  - Am J Physiol, 1997   **Pentoxifylline blocks hepatic stellate cell activation independently of phosphodiesterase inhibitory activity.** .
    Activated, but not quiescent, hepatic stellate cells lipocytes have a high level of collagen type I and smooth muscle **actin** SMA gene expression.
    Therefore, stellate cell activation is a critical step in hepatic fibrosis.
    The mechanisms leading to stellate cell activation in vivo are unknown.
    The characteristic hepatic oxidative stress cascade induced in rats by CCl4 markedly stimulated stellate cell entry into S phase, nuclear factor NF kappa B activity, and c myb expression.
    These changes were prevented by pentoxifylline, which also decreased CCl4 induced hepatic injury.
    As expected, cAMP mediated phosphorylation of **CREB** Ser133 was induced in vivo in stellate cells by pentoxifylline but not by its metabolite 5, an N 1 carboxypropyl derivative, which lacks phosphodiesterase inhibitory activity.
    Stellate cell nuclear extracts from CCl4 treated, but not from control, animals formed a complex with the critical promoter E box of the alpha SMA gene, which was disrupted by c myb antibodies and competed with by c myb cognate DNA.
    Treatment with pentoxifylline or metabolite 5 prevented the molecular abnormalities characteristic of stellate cell activation induced by CCl4.
    These results suggest that induction of c myb plays an important role in the in vivo activation of stellate cells.
    Pentoxifylline blocks stellate cell activation in vivo independently of its inhibitory effects on phosphodiesterases by interfering with the oxidative stress cascade and the activation of NF kappa B and c myb.
